# Psychological Inflexibility and Geriatric Primary Care: Transforming Peri-Urban and Rural Aging

**DOI:** 10.1093/geroni/igab046.3046

**Published:** 2021-12-17

**Authors:** Lindsey Jacobs, Rebecca Allen, Timothy Ly, John Bell, Dana Carroll, Anne Halli-Tierney

**Affiliations:** 1 The University of Alabama, The University of Alabama, Alabama, United States; 2 University of Alabama, Tuscaloosa, Alabama, United States; 3 University of Alabama, University of Alabama, Alabama, United States; 4 The University of Alabama, Tuscaloosa, Alabama, United States; 5 Auburn University Harrison School of Pharmacy, Tuscaloosa, Alabama, United States

## Abstract

Based on the acceptance and commitment therapy (ACT) framework, human suffering is thought to be caused by psychological inflexibility. Psychological inflexibility is characterized by rigid avoidance of unpleasant experiences, fusion with unhelpful thoughts, lack of contact with the present moment, fusion with a narrow self-narrative, and lack of clarity and contact with one’s core values in life. Psychological inflexibility captures the unhelpful or unworkable ways in which individuals respond to emotional discomfort. Research using samples of adults under age 65 indicate that psychological inflexibility is associated with poorer quality of life and mental well-being; however, the literature on psychological inflexibility in older adults is limited. Patients (N=129) ages 65 and older presenting to a Geriatric Primary Care clinic in the Deep South completed measures of depression, anxiety, subjective health literacy, and psychological inflexibility. Our team used the Acceptance and Action Questionnaire-II (AAQ-II), which is the most commonly used measure of psychological inflexibility. Anxiety (r = 0.66, p < .001) and depression (r = 0.70, p < .001) were moderately correlated with psychological inflexibility, which is consistent with the existing literature on psychological inflexibility in adults under the age of 65. Subjective health literacy significantly predicted psychological inflexibility, b = –.058, t(127) = -4.07, p < .001. This finding provides additional support for the importance of increasing health literacy among older adults in the Deep South, as it has implications in level of psychological flexibility and, thus, quality of life and mental well-being.

